# Safe by design (SbD) and nanotechnology: a much-discussed topic with a prudence?

**DOI:** 10.1186/s12989-021-00423-0

**Published:** 2021-08-23

**Authors:** Mary Gulumian, Flemming R. Cassee

**Affiliations:** 1 National Institute for Occupational Health, Johannesburg, South Africa; 2 University of the Witwatersrand, Johannesburg, South Africa; 3 Water research group, Northwest University, Potchefstroom, South Africa; 4 National Institute for Public Health and the Environment, PObox 1, 3720 BA Bilthoven, the Netherlands; 5 Institute for Risk Assessment Sciences, Utrecht University, Utrecht, The Netherlands

## Abstract

Safe-by-Design (SbD) has been put forward as a concept to assure that only safe nanomaterials will reach the market and that safety aspects have already been considered in a very early stage of the innovation process. In practice, several laboratory test have been proposed to screen newly developed nanomaterials and nano-enabled products to assess their hazardous nature. These tests need to have sufficient predictive power for possible adverse effects on human health, not only due to acute (peak) exposures, but also for long-term (low dose) exposures as these materials may accumulate over time in organs and tissues.

The concept of Safe-by-Design (SbD) has been implemented in drug design [1], crop breeding innovation [2], biotechnology [3], and in engineering disciplines [4]. It is introduced to identify risks, and minimize or even eliminate these during the early stages of the technological development [5]. Lessons learned from these industries on the SbD concepts and methods applied in hazard, exposure, and risk assessment, and eventually to risk management along its innovation value chain, are encouraged to apply to nanotechnology and the development of advanced and smart materials. The concept of SbD envisages including safety into innovation from the design phases and early development of a new nanomaterial or nano-enabled products, instead of conducting toxicity assessments only after nanomaterials reach the market [6, 7]. These publications have emphasized the importance of addressing the potential health and safety risks at different stages of the development of nanomaterials. This includes the synthesis (safe material/product), processing, handling, and incorporation into products (safe production) stages, and finally to their disposal at end-of-life cycle (safe use and end of life). For the application of this concept, a comprehensive approach was recommended for all the specified stages of SbD.

For the early development and design phases of a new nanomaterial or nano-enabled products, an inventory of testing strategies is made to provide knowledge on the properties that make a nanomaterial or nanoproduct safe. Data requirements at this stage include basic toxicological information to establish the relationship between designed nanomaterial properties, their interactions with biological systems, and effects at the cellular and molecular level [6]. Moreover, to address the potential health risks of nanomaterials during the product design (idea) stage it is necessary to identify the intrinsic hazards and to include the knowledge of the role of size, surface, and shape, and functionalization of nanomaterials [8]. Most recently, existing regulatory accepted toxicity tests, applicable for safety screening of nanomaterials, were critically reviewed with a conclusion that no recommendations for specific experimental assays could be given [7]. The most suitable to be used depend on the type of nanomaterial and the expected exposure scenario, transformation, translocation and on the potential target organs.

For the design of safe(r) nanomaterials in the manufacturing processes, it is necessary to design work methods and operations, processes, equipment, tools, products, materials, new technologies, and the organization of work in such a way that risk for exposure is minimized. Moreover, process safety also deals with all the accident scenarios that might be encountered during processing and the possible injuries to workers and damage to the environment. Again, the importance of reduction of each hazard was stressed with recommendations of using high-throughput screening and evaluation techniques to assess the toxic potency of the materials. The point has also been stressed that the most efficient means of preventing high-risk exposure is to substitute any material with a less hazardous one and therefore by designing nanomaterials with lower toxicity to decrease the hazard [9]. To assess the risk of such accidents, one has to know the physicochemical, toxicological and ecotoxicological hazards of the substances involved.

Finally, to address safe use and end-of life, minimizing exposure has been proposed to minimise the adverse effects associated with exposure to the nanomaterials through their entire use life, recycling and disposal [10]. It has been suggested that when a product has been made as safe as is possible in the first stage of SbD, this will facilitate, in the last stage of SbD, the evaluation and determination of any potential restrictions on the use of a specific hazardous nanomaterial, and thus minimise the associated adverse effects through their entire use life [11].

It is therefore evident that SbD concept involves the design of the nanomaterials being synthesized, the design of processes involved in their production and finally the safe design of consumer products to reduce nanomaterial release from these products. It is also evident, from the above cited publications that the decisions made in the design stage of the nanomaterials synthesis will determine, at least in part, how hazardous the nanomaterials are that are being produced or incorporated into (consumer) products. With the acknowledgment of the importance of hazard identification of the nanomaterials being synthesised, it brings us to the crucial question of how to determine the hazardous nature of nanomaterials that are being synthesised through SbD.

Within NANoREG (https://www.rivm.nl/en/about-rivm/mission-and-strategy/international-affairs/international-projects/nanoreg) and other European projects, including NanoValid (http://www.nanovalid.eu/), and Nanogenotox (http://www.nanogenotox.eu/), standard toxicological protocols have been adapted to the assessment of the toxicity of nanomaterials. Commonly used assays to assess cell viability include MTT, XTT, MTS and WST (that determine metabolic activity based on reducing tetrazolium dye) and also Alamar blue and neutral red. Assays used to assess genotoxicity/carcinogenicity include Comet assay, micronucleus, in vitro mammalian cell gene mutation test and cell transformation (CTA) assay. Finally, to assess the generation of reactive oxygen species, include 2′-7′-dichlorofluorescin (DCFH) and 2,2-diphenyl-1-picryl-hydrazyl-hydrate (DPPH) free radical scavenging assays.

Some of the aforementioned assays have been subject to inter-laboratory comparisons to assess the reproducibility of the assay, e.g. the round robin exercises performed within NANoREG [12]. There are number of concerns in the tests recommended for hazard identification in the early development and design phases of a new nanomaterial or nano-enabled products of the SbD.

In several cases the nature of the nanomaterials may interfere with most of the listed detection methodologies [13]. Additionally, it is essential to test for endotoxin contamination before studying the immunotoxicity of nanomaterials in vitro. Hence, the development and use of validated assays is still a critical issue that needs to be addressed prior to their implementation to confirm or negate the toxicity of nanomaterials in this initial stage of SbD [14]. Therefore, interference will have a major impact on the hazard identification of nanomaterials. Toxicity tests must be reliable and free of interference by nanomaterials, which is at present still a major concern related with nanomaterial hazard identification.

In addition, short-term in vitro toxicity tests may not be able to predict long-term effects or even predict the complex response in organs such as lung and gastrointestinal tract. For example, nanomaterial biodurability may be assessed through the determination of dissolution rate constants. The latter will provide an indication of their biodurability, defined as the ability to resist chemical/biochemical alteration, which is a significant contributor to biopersistence. Biopersistence of nanomaterials will contribute to their long-term effects, as when the clearance rate is slower than the accumulative rate, they will accumulate in the relevant organs. Prolonged organ retention of nanomaterials may eventually lead to persistent inflammation, which is considered to lead to adverse outcomes such as fibrosis and tumours. The involvement of lysosomal membrane permeabilisation (LMP) and NLRP3 inflammasome activation has recently been emphasized [15]. As lysosomal dysfunction has been involved in disease pathogenesis, the association of nanoparticle exposure and lysosomal dysfunction and inflammasome activation may have relevance to nanomaterial-induced chronic toxicity. This may give the opportunity to use these parameters to investigate the long-term effects of nanomaterials [16].

Moreover, the importance of persistent inflammation has recently been recognised. New in vitro tests may be implemented to predict the long-term effects of nanomaterials though validation has not yet happened [17]. Current testing guidelines may also not be suitable to accommodate testing of, for example, immunotoxic effects, such as complement activation-related pseudoallergy, myelosuppression, inflammasome activation, and hypersensitivity which are not readily detected. At present, effort is put into demonstrating the predictive value of simple or even complex in vitro models for human and environmental health effects, and this is a crucial part of getting the test accepted in a regulatory framework and successfully implemented in SbD approaches. Decades of experience in particle and fibre toxicology has taught us that effects of solid materials are not only related to their chemical nature but also to the physical aspects like size, shape, and surface area. This is at present less well incorporated in the tests that are proposed for hazard screening and risk estimation of nano, smart and advanced materials.

In conclusion, SbD for both safe production and end-of life cycle, which ensure protection of the workers, consumers, and the environment, are achievable goals. The success of the SbD in the design stage may, however, depend on the testing strategies implemented to assess the hazardous nature of the nanomaterials and nano-enabled products being synthesised. The question therefore that needs to be asked is: are the appropriate testing methods being implemented, which are free of interference by nanomaterials, not only focussing on acute toxicity but also predictive enough for their (long-term) effects? The latter aspect ought to be addressed when publishing the research in scientific journals.

## Data Availability

Not applicable.

## References

[1] Schmutz M, Borges O, Jesus S, Borchard G, Perale G, Zinn M, et al. A methodological safe-by-design approach for the development of nanomedicines. Front Bioeng Biotechnol. Front. Bioeng. Biotechnol., 02 April 2020. 10.3389/fbioe.2020.00258. 10.3389/fbioe.2020.00258 PMC7145422 32300587

[2] Van der Berg JP, Kleter GA, Battaglia E, Bouwman LMS, Kok EJ (2020). Application of the safe-by-design concept in crop breeding innovation. Int J Environ Res Public Health.

[3] Bouchaut B, Asveld L (2020). Safe-by-design: stakeholders’ perceptions and expectations of how to Deal with uncertain risks of emerging biotechnologies in the Netherlands. Risk Anal.

[4] Steve W (2018). Briefing: network rail safe by design: buildings and civils working group, UK. Proceedings of the Institution of Civil Engineers - Forensic Engineering.

[5] Hale A, Kirwan B, Kjellén U (2007). Safe by design: where are we now?. Saf Sci.

[6] Kraegeloh A, Suarez-Merino B, Sluijters T, Micheletti C. Implementation of safe-by-Design for Nanomaterial Development and Safe Innovation: why we need a comprehensive approach. Nanomaterials (Basel). 2018;8(4). 10.3390/nano8040239. 10.3390/nano8040239 PMC5923569 29661997

[7] Dekkers S, Wijnhoven SWP, Braakhuis HM, Soeteman-Hernandez LG, Sips AJAM, Tavernaro I, Kraegeloh A, Noorlander CW (2020). Safe-by-design part I: proposal for nanospecific human health safety aspects needed along the innovation process. NanoImpact.

[8] Morose G (2010). The 5 principles of “Design for Safer Nanotechnology”. J Clean Prod.

[9] Geraci C, Heidel D, Sayes C, Hodson L, Schulte P, Eastlake A, Brenner S (2015). Perspectives on the design of safer nanomaterials and manufacturing processes. J Nanopart Res.

[10] Lin S, Yu T, Yu Z, Hu X, Yin D (2018). Nanomaterials safer-by-design: an environmental safety perspective. Adv Mater.

[11] Köhler AR, Som C (2014). Risk preventative innovation strategies for emerging technologies the cases of nano-textiles and smart textiles. Technovation.

[12] Nelissen I, Haase A, Anguissola S, Rocks L, Jacobs A, Willems H, Riebeling C, Luch A, Piret J-P, Toussaint O, Trouiller B, Lacroix G, Gutleb AC, Contal S, Diabaté S, Weiss C, Lozano-Fernández T, González-Fernández Á, Dusinska M, Huk A, Stone V, Kanase N, Nocuń M, Stępnik M, Meschini S, Ammendolia MG, Lewinski N, Riediker M, Venturini M, Benetti F, Topinka J, Brzicova T, Milani S, Rädler J, Salvati A, Dawson KA (2020). Improving quality in nanoparticle-induced cytotoxicity testing by a tiered inter-laboratory comparison study. Nanomaterials.

[13] Andraos C, Yu IJ, Gulumian M (2020). Interference: a much-neglected aspect in high-throughput screening of nanoparticles. Int J Toxicol.

[14] Giannakou C, Aimonen K, Bloois LV, Catalán J, Geertsma RE, Gremmer ER, de Jong WH, Keizers PH, Schwillens PL, Vandebriel RJ, Park MV (2019). Sensitive method for endotoxin determination in nanomedicinal product samples. Nanomedicine (Lond).

[15] Stern ST, Adiseshaiah PP, Crist RM (2012). Autophagy and lysosomal dysfunction as emerging mechanisms of nanomaterial toxicity. Part Fibre Toxicol.

[16] Chen RJ, Chen YY, Liao MY, Lee YH, Chen ZY, Yan SJ, et al. The current understanding of autophagy in nanomaterial toxicity and its implementation in safety assessment-related alternative testing strategies. Int J Mol Sci. 2020;21(7). 10.3390/ijms21072387 PMC7177614 32235610

[17] Kämpfer AAM, Urbán P, La Spina R, Jiménez IO, Kanase N, Stone V, Kinsner-Ovaskainen A (2020). Ongoing inflammation enhances the toxicity of engineered nanomaterials: application of an in vitro co-culture model of the healthy and inflamed intestine. Toxicol in Vitro.

